# Influencing factors of online products decision-making oriented to tourism economy under the guidance of consumer psychology

**DOI:** 10.3389/fpsyg.2022.950754

**Published:** 2022-08-03

**Authors:** Linlin Jin, Bin Hu

**Affiliations:** ^1^Tourism College of Zhejiang, Hangzhou, China; ^2^Department of Law, Zhejiang University City College, Hangzhou, China

**Keywords:** online travel products, tourism economy, online travel platform, user characteristics, consumer psychology

## Abstract

This work aims to increase the consumption of online tourism products and promote the development of the tourism economy. Based on this, it first analyses the Internet market under the guidance of consumer psychology. Then, the influencing factors of online product decision-making for the tourism economy are discussed. Finally, based on the above analysis, it discusses and evaluates the main factors affecting the consumption of online travel products. The research method of this work is set based on psychology so that it can analyze the psychological state of consumers more deeply and promote the development of the consumer market. The results show that the main factors affecting the consumption of online travel products include online travel platforms and user characteristics. Specifically, approximately 80% of users consume online travel products based on platform reviews, approximately 10% of users consume online travel products based on platform recommended content, and approximately 5% of users consume online travel products based on platform search content. Users vary mainly by age, gender, and region and have different preferences for different platforms. Among the four major platforms, Ctrip occupies the most consumers. The conclusion is that the main way to develop the tourism economy is to build a better online travel platform. At the same time, it is necessary to promote online tourism according to the characteristics of users and increase the marketing of online tourism products. This work not only provides a reference for promoting online tourism product marketing but also helps to promote the development of the tourism economy.

## Introduction

With the development of science and technology, the marketing method of tourism products is also changing and is gradually moving toward online marketing. In the embryonic stage of online marketing, developing online tourism platforms to attract more users is the main driving force to promote the development of the tourism economy (Talwar et al., 2020). Major online tourism platforms, as the main support for online tourism product marketing, also have an important impact on the development of the online tourism economy. Through research, the factors influencing online product decision-making are found, and the improvement of online tourism platform construction plays an important role in promoting the development of the tourism economy (Giroux et al., 2022).

Chang et al. (2020) pointed out that with the rapid development and popularization of the Internet in China, the number of netizens continues to grow, and the characteristic structure of netizens is also changing accordingly. How to identify potential customers of a company from a large group of netizens and analyze their psychological and behavioral characteristics is the primary task of online marketing for companies (Chang et al., 2020). Marine-Roig and Huertas (2020) pointed out that consumer psychology research is an important support system for industrial design research. However, most of the research in the field of industrial design is still in the narrow sense of humanized design, that is, the discussion of the relationship between products and people, and the focus is on the microman–machine interface, which is not comprehensive. In this consumer-oriented era, design and psychology are increasingly closely related, and whether to pay attention to this connection has become an important part of product design. Products that can meet the needs and preferences of consumers must have more chances to win. The morphological design of products often plays a pivotal role in it (Marine-Roig and Huertas, 2020). Assaker (2020) pointed out that with the rapid development of the Internet in recent years, consumers are increasingly inclined to book travel products online. As an important form of online word of mouth, online user reviews have become an important reference for consumers to make purchasing decisions. User comments and replies have a significant impact on the sales of products and services, so many companies have begun to respond positively to users' online comments to improve users' favorable impression of the company's services and remedy the negative impact of negative comments (Assaker, 2020). Huang and Mou (2021) proposed that the rapid development of network technology makes the Internet and various fields of the economy and society integrate with each other, changing people's consumption patterns. As an important consumption field, tourism has changed due to the Internet in many aspects from product design, marketing promotion, industrial composition, profit model, etc. “Internet + tourism” shows vigorous vitality. According to the “2018 China Online Travel Industry Research Report” released by iResearch, the transaction scale of China's online travel market exceeded 1.48 trillion yuan in 2018, a year-on-year increase of 26.3% compared with 1.17 trillion yuan in 2017, setting a new record. The vigorous development of the online travel industry not only has an impact on the consumption patterns of travelers but also changes the dissemination of travel information. A large amount of user-created content has become an important source of information and decision-making basis for consumers. Therefore, understanding the perception attitude, usage habits, and influencing factors of travel consumers toward the Internet Word of Mouth (IWOM) is of great research significance and an important research topic for expanding the application of IWOM in the tourism industry (Huang and Mou, 2021). Le et al. (2022) pointed out that the competition in the online travel industry has intensified unprecedentedly, mainly in two aspects. First, the traditional business model is challenged. With the changes in the market environment and the rise of personalized tourism, more diversified business models have emerged. The new business model has more advantages in product innovation, service optimization, and price control and is more in line with consumers' consumption habits and travel needs. The traditional online travel agency model has been greatly challenged and even has a tendency to be surpassed in some segments, so there have also been unfair competition methods such as “price war”. Second, product suppliers are threatened. As the interesting relationship between online travel agencies and product suppliers becomes increasingly difficult to reconcile, leading to the rise of the supplier direct sales model, resulting in a more complex relationship between online travel agencies and suppliers. One side is cooperating out of desperation, and the other side is competing. The increasingly tense relationship makes the operation of the online tourism industry chain increasingly difficult (Le et al., 2022). It can be concluded from the above research that the current consumption of online travel products has become the main development goal of social tourism, and this goal can also effectively promote the development of the tourism economy and then provide an impetus for social development. However, the current research has not addressed the influencing factors of the online tourism industry, so evaluating online product consumption through research plays an important role in the development of the tourism industry.

Based on this, this work first discusses the online market under the guidance of consumer psychology, then discusses online product decision-making oriented to the tourism economy, and finally explores and evaluates the consumption data of online tourism products. The novelty of this work is that it has carried out in-depth research on users from the perspective of psychology and carried out research on the consumer market from the perspective of basic factors, which improves the rationality and practicability of the research. This work not only provides a reference for online tourism product marketing but also contributes to the comprehensive development of the tourism economy.

## Research theory and methods

### Online market under the guidance of consumer psychology

Consumer psychology refers to the psychological activities of consumers (Argo, 2020). Psychological activity is the reflection of the human brain on objective things or external stimuli. As a special function of the human brain, it is in an inner hidden state and does not have a directly observable form (Jain and Weiten, 2020), but it can govern human behavior, determining what people do, what they do not do, and how they do it. The deeper explanation of consumer psychology is a series of psychological activities of consumers in the process of purchasing, using, and consuming commodities. As a specific group, consumers must have some common characteristics of human beings. They all have thoughts, feelings, desires, passions, and interests. All these characteristics constitute human psychology, that is, psychological activities or psychological phenomena. In a variety of consumption activities, consumers will produce a series of psychological activities (Maglio, 2020). Different consumers have distinct psychological activities. Understanding consumer psychology and personality characteristics can better guide marketing activities and, on this basis, stimulate and induce consumers' purchasing power. By providing a variety of appropriate services and solving various problems in buying activities, merchants can sell more goods and improve consumer satisfaction with goods. Consumption, as an objective economic phenomenon in human society, is the behavior of people paying for material products and labor services in social and economic activities (Furth-Matzkin and Sommers, 2020).

However, consumption cannot be completed without certain psychological motivation. So-called consumer purchasing motivation refers to the desire or intention of consumers to buy a certain commodity or service to meet their own needs. It is the internal motivation that can cause consumers to buy a certain commodity or service (Wedel and Dong, 2020). Purchasing motivation is the direct reason that produces consumption demand and causes consumer buying behavior. Compared with consumer demand, motivation is clearer and more specifically in relation to consumer behavior. Due to the diversity of consumers' needs, the complexity of consumers' purchasing motivation is conceivable. In real life, it can be generally divided into physiological buying motivation and psychological buying motivation. Physiological purchase motivation is the purchase motivation triggered by consumers to maintain living organisms. Such purchase motivation is based on physiological needs and has the characteristics of regularity, universality, repetition, habituation, and dominance (Ozanne et al., 2022). Psychological purchasing motivation refers to the purchasing motivation caused by consumers' cognitive, emotional, will, and other psychological processes. The psychological factors of consumers are the root of their psychological purchasing motivation, such as emotional motivation, rational motivation, and patronage motivation, which are all important factors that determine consumers' purchasing strategies (Schwarz, 2021).

Currently, the online market is the main consumer market, while the traditional offline consumer market and online market are two completely different platforms. Offline consumption platforms are more real for consumers, that is, consumers can access real products when they make offline consumption and have strong confidence in their consumption actions. However, online consumption platforms are completely different; that is, on online consumption platforms, users are exposed to fake products, so users generally have low confidence in online shopping. With the development of science and technology, online shopping has become the main form of social consumption (Davis and Pechmann, 2020). Therefore, it is the main trend that industries promote their own development through online consumption platforms. Similarly, society heads forward by stimulating social consumption.

### Tourism economy-oriented online products sale

Travel means going out, that is, from a place to b place, for sightseeing and entertainment (Sharma et al., 2022). In current society, tourism has become one of the main forces promoting social and economic development (Assaker et al., 2020). The main products of tourism consumption are not tourist locations or scenic spots but various products around tourism, such as current tourist souvenirs, scenic specialties, and delicacies (Angeloni and Rossi, 2021).

**Figure 1:**
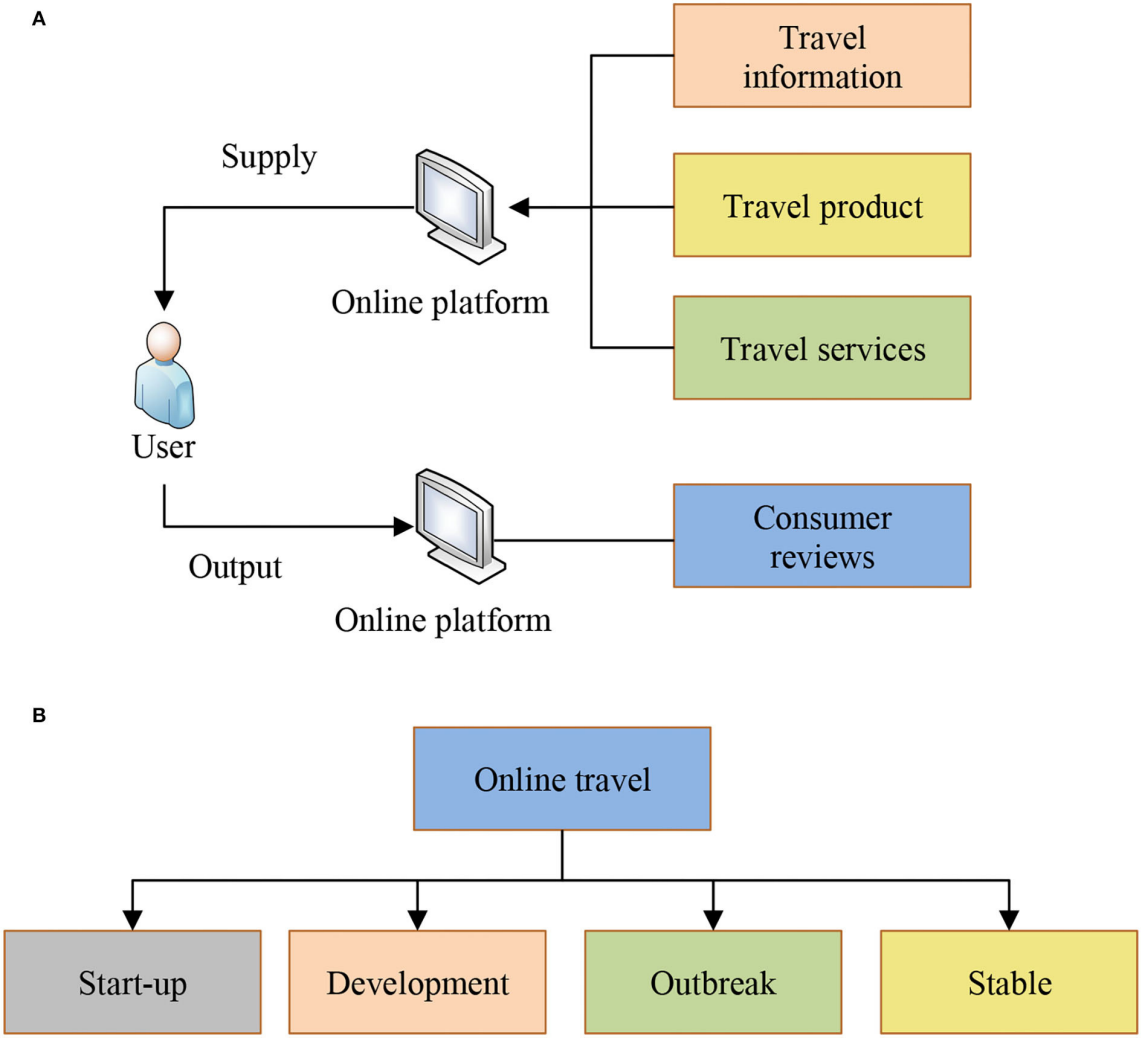
Main characteristics of online travel. **(A)** is the procedure of online travel, and **(B)** is the development history of online travel.

Online travel refers to an industry that provides consumers with travel information, products, and related services through the Internet, and tourists can share travel experience through the Internet. From the perspective of transaction scale, the transaction scale of China's online travel market in 2018 was 1,512.24 billion yuan, with an increase of 29.0% compared with 2017. The projected growth rate for 2019 also reached 18.8%, with the transaction scale reaching 1,79,654 billion yuan. The rise of online travel in the late 1990s roughly experienced the initial stage, development stage, outbreak stage, and stable stage. The initial stage was the “commission model” of air ticket booking + hotel booking, that is, the website in cooperation with hotels and airlines, to provide users with travel information, air ticket booking, and hotel booking business. In this stage, hotel booking agents were the main source of income. The development period was characterized by online travel product agencies. In other words, a variety of offline tourism products emerge, and platform business and online booking business coexist in the market, forming a diversified system (Zhang et al., 2021). In this stage, air ticket agents developed rapidly and became the main source of income. The breakout period featured vertical market segments with the influx of a large amount of capital (Tuniu providing tourist attractions and travel route design, social travel sites such as Hornet's Nest and Tujia in the field of non-standard accommodation). In this stage, merchants acquire travel agencies and establish offline outlets to integrate offline industrial chain resources and enhance their control over offline resources. In a stable period, the basic integration of resources has been completed, and an industry pattern has formed (Huang and Lan, 2021). At the same time, the Internet dividend gradually disappears, the cost of introducing new users increases, and merchants have to mine the value of old users. Figure 1 shows the main characteristics of online travel.

As shown in Figure 1, the development of the tourism economy is the main method of social tourism development at present, and its development also has many influencing factors. Analyzing its influencing factors plays an important role in developing an online tourism mode and promoting the development of the online tourism economy.

### Sources of research data

First, the basic information of most of the Southeast Asian tourism products launched by the website is obtained through the web crawler method. The data of four online travel network platforms, such as Ctrip Travel, Qunar Travel, Tongcheng Travel, and Fliggy Travel, were obtained, and a total of 600 different merchants released basic product information. Table 1 shows the basic information of four online travel network platforms, such as Ctrip Travel, Qunar Travel, Tongcheng Travel, and Fliggy Travel.

**Table 1:** Basic information of online travel network platforms.

**Travel platform**	**Founded time**	**Amount of users**	**2019 tourism ranking index**
Xiecheng	1999	135.2 million	100
Qunar	2005	34.1 million	45.13
Tongcheng	2004	31.3 million	20.25
Feizhu	2016	200.1 million	41.15

Based on the content of Table 1, consumers make purchase behaviors under the guidance of consumer psychology (Wicaksono and Maharani, 2020). In online travel product marketing, the online travel platform is the main factor that affects its development, and reviews are one of the important reasons users choose products. Analysis of user reviews reveals the characteristics of buyers' consumption behavior and their inner thoughts and feelings about products to continuously improve product quality and attract more users to purchase from the perspective of user experience (Choi et al., 2022). Moreover, as the main body of online travel product consumption, different groups of people have an important influence on online travel product consumption. The different groups mainly include region, age, and sex. First, the region is the main factor that affects the user's tourism consumption because the economic characteristics of different regions are different, so the consumption ability of the user's consumption concept is different. Therefore, users in different regions have different degrees of online tourism product consumption, so regions have an important impact on the development of the tourism economy (Shi and Hu, 2021). Second, as the main factor affecting user consumption, age also has an important impact on the development of the tourism economy. Finally, gender is also the main factor that currently affects user consumption because different gender consumption concepts and consumption orientations will also be different (Liang et al., 2021). In summary, this work will contribute to promoting the development of the tourism economy by researching and evaluating the factors influencing the decision-making of online tourism products by the classification of online tourism platforms, user types, and tourism types.

The Support Vector Machine (SVM) algorithm is employed for data analysis, which is a learning system using a linear function hypothesis space in a high-dimensional feature space. It is trained by a learning algorithm under optimization theory (Mohammadi et al., 2021). Its calculation equation is as follows:


(1) $$\begin{array}{r} w\cdot x+b=0 \end{array}$$

where *w* represents the normal vector of the hyperplane, *b* represents the constant term of the hyperplane, and *x* represents the data sample, whose main task is to calculate the optimal *w* and *b*. Let the optimal *w* and *b* be *w*_0_ and *b*_0_, expressed as follows.


(2) $$\begin{array}{r} w_{0}\cdot x+b_{0}=0 \end{array}$$

The label of the test set is expressed as follows:


(3) $$\begin{array}{r} t_{i_{-}}label\;=sgn\left(w_{0}\cdot t_{i}+b_{0}\right) \end{array}$$

where *t*_*i*_ represents a collection of tests. Suppose the data point is (*x*_*i*_, *y*_*i*_), and then the support vector satisfies the following conditions:


(4) $$\begin{array}{rcl} w\cdot x_{i}+b & = & -1,y_{i}=-1 \end{array}$$

(5) $$\begin{array}{rcl} w\cdot x_{i}+b & = & 1,y_{i}=+1 \end{array}$$

The above equations show the standard of positive and negative cases, and then the interval between the two is expressed as follows:


(6) $$\begin{array}{r} dis=\frac{w}{∥w∥}\cdot \left(x_{1}-x_{2}\right) \end{array}$$

The above problems can be solved using the Lagrange function, calculated as follows:


(7) $$\begin{array}{c} J(w,b,a)=\frac{1}{2}w^{T}w-\sum_{i=1}^{N}a_{i}[y_{i}(w\cdot x_{i}+b)-1] \end{array}$$

where *a* represents the auxiliary non-negative variable, *T* represents the bias matrix, *i* represents the sample, and *N* represents the total sample size. The optimal solution can be calculated as follows:


(8) $$w_{0}\;=\;\sum_{i=1}^{N}a_{i}^{{}^{*}}y_{i}x$$

(9) $$\begin{array}{rcl} b_{0} & = & 1-w_{0}x^{\left(s\right)},y^{\left(s\right)}=1 \end{array}$$

**Figure 2:**
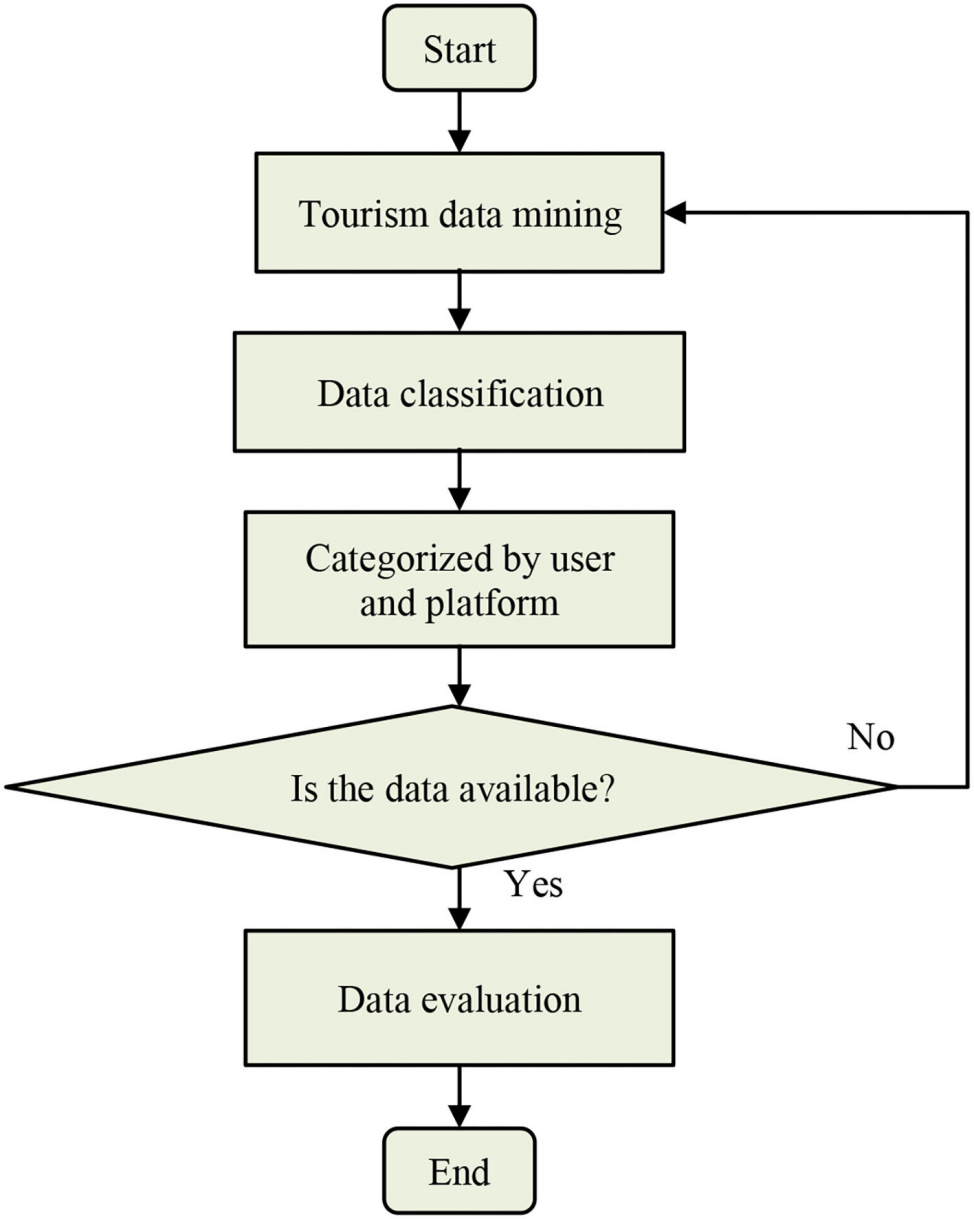
The basic flow of data setting.

After the above calculation, the data will be classified and evaluated to determine the factors that ultimately affect the development of the tourism economy (Otchere et al., 2021). Figure 2 shows the specific process of data evaluation and classification by the SVM algorithm.

As shown in Figure 2, when the data are output as the optimal solution, they become the main basic information. After certain analysis and evaluation, the main role of the data is determined.

## Influencing factors on decision-making of online travel products

### Influence of online travel platform on decision-making of online travel products

**Figure 3:**
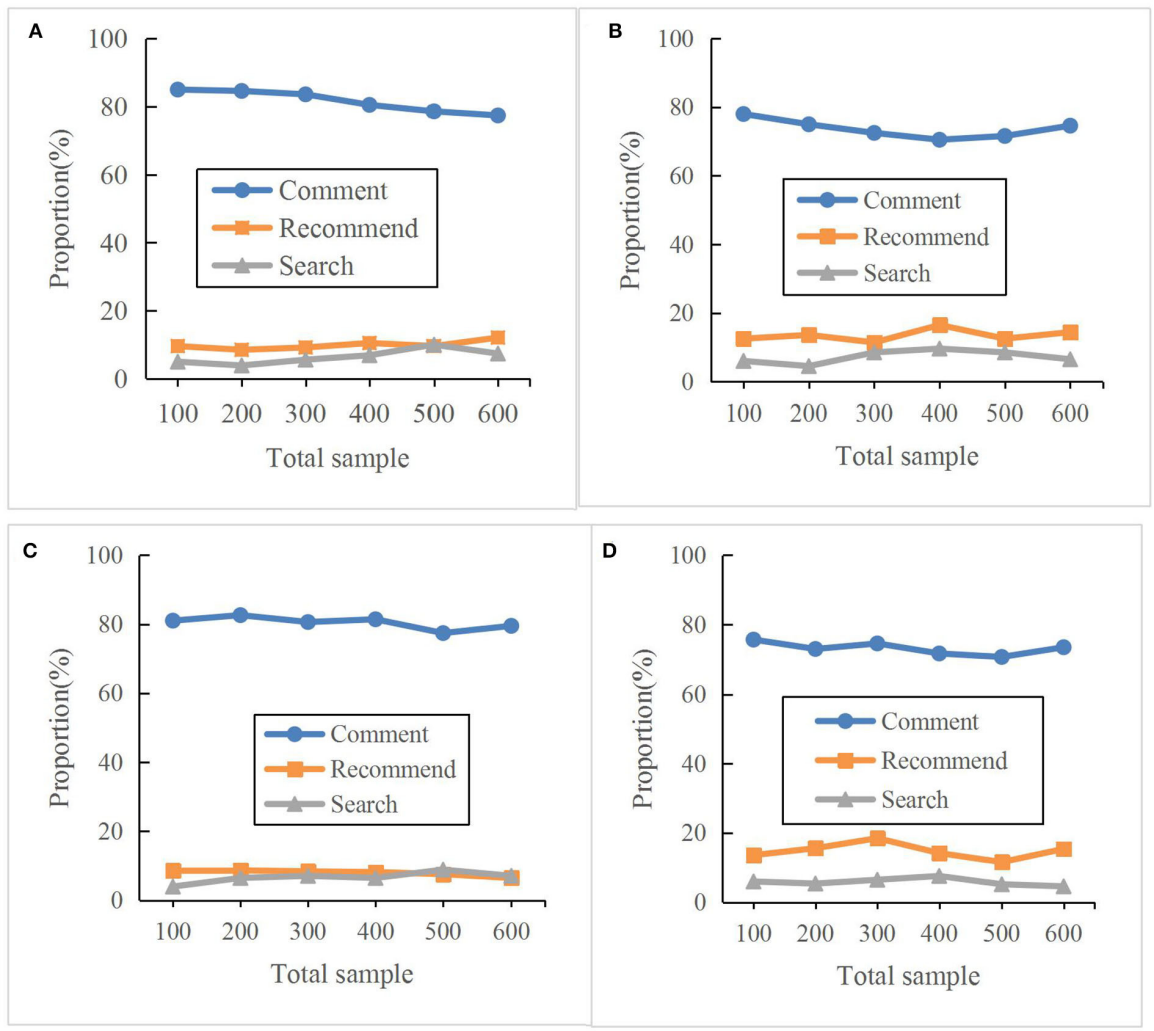
The impact of the four platforms on the decision-making of online travel products. **(A)** Ctrip; **(B)** Qvnaer; **(C)** TravelGo; **(D)** Fliggy.

The online travel platform is the main development channel to support the sales of online travel products at present. Analyzing the impact of tourism platforms on the tourism economy through data is an important part of our research. Factors that have a significant impact on online travel platforms include comments and the main types of services available on the platform. Figure 3 shows the effects of the four platforms on the sales of tourism products.

Of the four platforms, users buying products according to the comments account for the largest proportion. Specifically, users who buy online travel products according to the comments on the platform account for approximately 80%. Users who buy online travel products according to the recommended contents on the platform account for approximately 10%. Users who buy online travel products based on the search content in the platform account for approximately 5%. It is noted that the most important basis for users to buy online travel products is the comments on the platform.

### Influence of users on decision-making of online travel products

**Figure 4:**
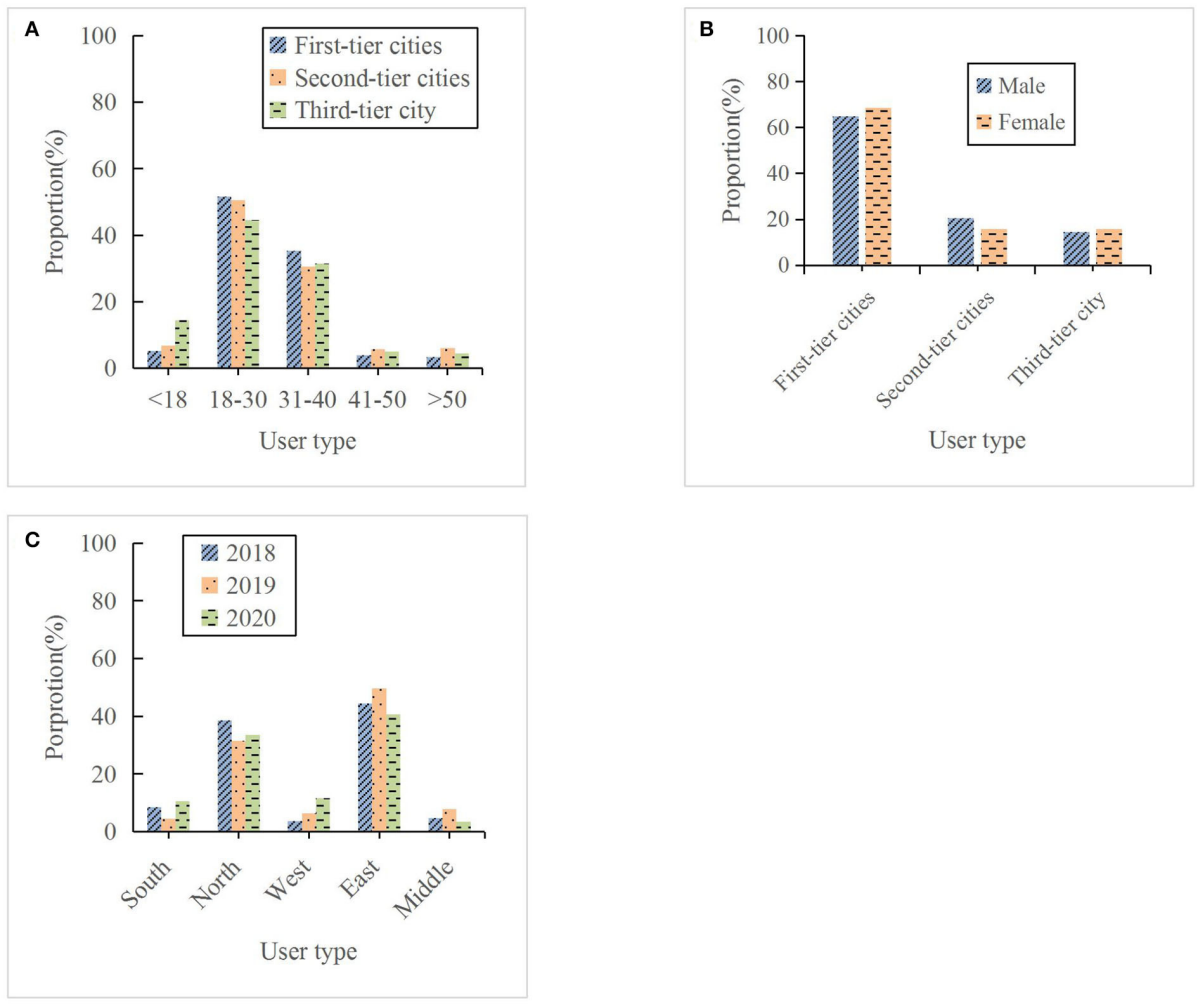
Consumption of online travel products of users on different platforms. **(A)** by age, **(B)** by gender, and **(C)** by region.

As the main participants in online travel product consumption, users have a great influence on the decision-making of online travel products. Users mainly differ by region, age, and gender. Figure 4 displays the consumption status of online travel products of users on different platforms.

**Figure 5:**
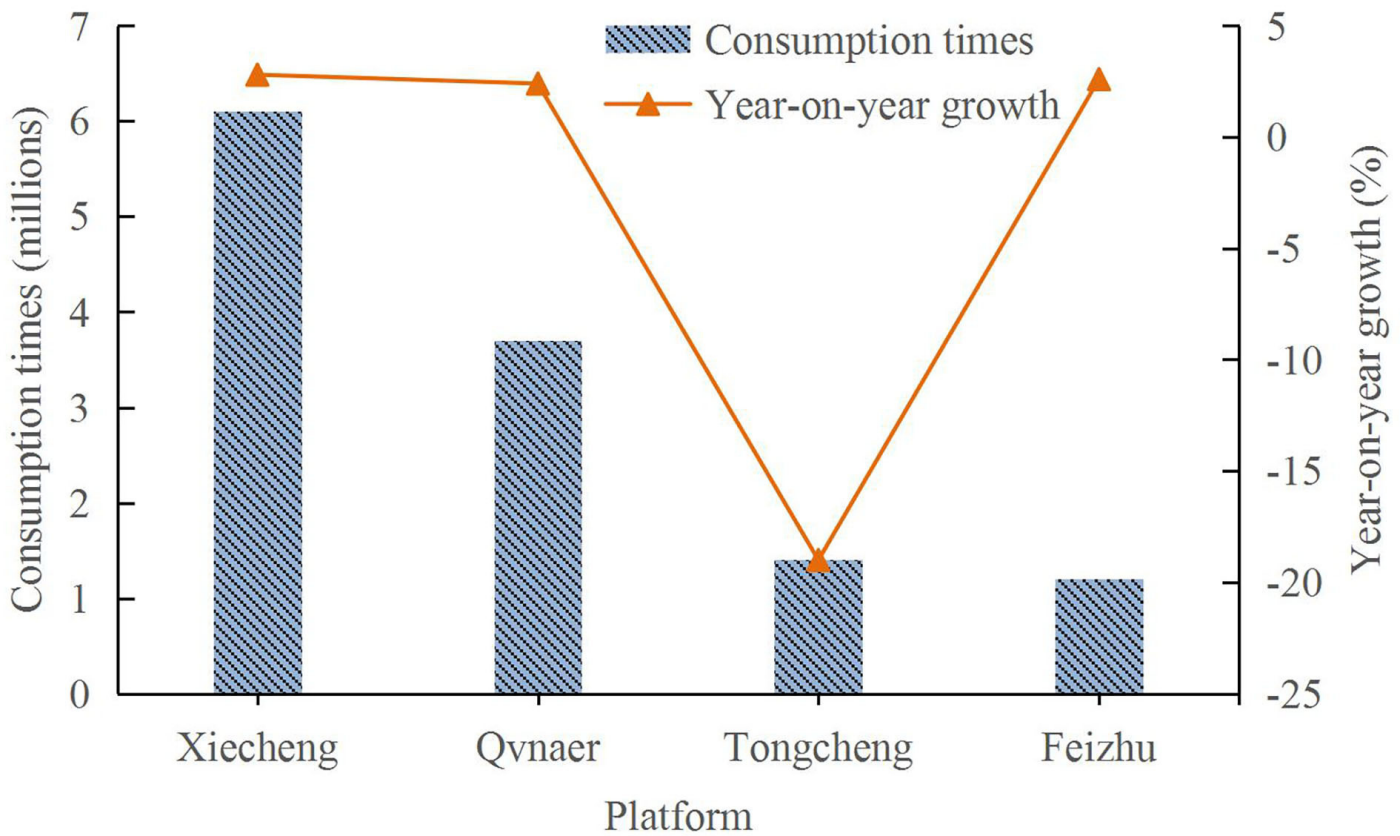
Consumption frequencies of users on different network platforms.

As shown in Figure 4, the proportion of online travel product consumption by different users will vary greatly. Among them, in the age classification, the users aged 18–30 are the most consumed online travel products, and the users aged 41–50 and over 50 are the least. In terms of gender classification, women who consume more online travel products are women, and men who consume less are men. In the regional classification, the users in the east are the largest consumers of online travel products, and the users in the west and the center are the smallest. Then, this work compares the consumption behavior of users from 2018 to 2020, reflecting the consumption status of online platforms under normal circumstances and under the influence of COVID-19, to more comprehensively analyze the service requirements of online travel platforms and improve the efficiency of online travel services. Figure 5 shows the online consumption status of users on different travel platforms in 2020.

Different network platforms also have a certain impact on user consumption. Of them, Ctrip has the largest number of users, and the number of users in 2020 will be approximately 60 million, while Fliggy has the lowest number of users, and the number of users in 2020 will be approximately 10 million. Ctrip has the highest year-on-year growth rate of 2.8%, while TravelGo has the lowest year-on-year growth rate of −19%. Different network platforms also have an important impact on the users' consumption of online travel products.

## Conclusions

With the development of network technology, online consumption has become the main form of consumption today, as has travel products. To increase the sales of online tourism products and promote the development of the tourism economy, the development of online tourism and the form of the tourism economy are discussed first. Then, this work explores the factors that affect the sales of online travel products. Finally, these factors are evaluated based on the data results.

The results of this work show that, in the research on the basis of user consumption, the number of users who consume online travel products according to the reviews on the platform is very large. The number of users who consume online travel products according to the recommended content in the platform is very small. In addition, the number of users who consume online travel products according to the search content in the platform is the lowest. Second, in the age classification, the users aged 18–30 who consume the most online travel products are the users aged 41–50, and those over 50 years old consume the least. In terms of gender classification, women who consume more online travel products are women, and men who consume less are men. In the regional classification, the users in the east are the largest consumers of online travel products, and the users in the west and the center are the smallest. In different online travel platforms, the number of consumer users is also very different. Therefore, according to the promotion of the development of online tourism, it must do a good job in the service of the platform first; second, it must carry out different online tourism promotions according to the type of users; and finally, it must strengthen the construction of the online tourism platform to attract the users' consumption. Although this work provides more accurate research results of influencing factors, the overall influencing factors included are not comprehensive enough, so future research will expand the exploration of factors affecting online tourism.

## Data Availability Statement

The datasets presented in this study can be found in online repositories. The names of the repository/repositories and accession number(s) can be found in the article/supplementary material.

## Author contributions

LJ and BH conceived and designed this study, analysis the data, and also contributed to the writing of this whole manuscript. Both authors contributed to the article and approved the submitted version.

## Funding

This work was supported by Hangzhou Key Research Base Project of Philosophy and Social Sciences.

## Conflict of interest

The authors declare that the research was conducted in the absence of any commercial or financial relationships that could be construed as a potential conflict of interest.

## Publisher's note

All claims expressed in this article are solely those of the authors and do not necessarily represent those of their affiliated organizations, or those of the publisher, the editors and the reviewers. Any product that may be evaluated in this article, or claim that may be made by its manufacturer, is not guaranteed or endorsed by the publisher.
